# Phase behavior of π-conjugated polymer and non-fullerene acceptor (PTB7-Th:ITIC) solutions and blends

**DOI:** 10.1038/s41598-022-25476-9

**Published:** 2022-12-02

**Authors:** Jung Yong Kim, Pawel Jarka, Barbara Hajduk, Henryk Bednarski, Urszula Szeluga, Tomasz Tański

**Affiliations:** 1 Department of Materials Science and Engineering, Adama Science and Technology University, P.O. Box 1888, Adama, Ethiopia; 2 Center of Advanced Materials Science and Engineering, Adama Science and Technology University, P.O. Box 1888, Adama, Ethiopia; 3 Institute of Engineering Materials and Biomaterials, Faculty of Mechanical Engineering, Silesian University of Technology, 44-100 Gliwice, Poland; 4 Centre of Polymer and Carbon Materials, Polish Academy of Sciences, M. Curie-Skłodowska 34 Street, 41-819 Zabrze, Poland

**Keywords:** Chemistry, Energy science and technology, Materials science, Physics

## Abstract

Phase diagrams of ternary π-bonded polymer (PTB7-Th) solutions were constructed as a function of molecular weight, temperature, and electron acceptor species (ITIC, PC_61_BM and PC_71_BM). For this purpose, the Flory–Huggins lattice theory was employed with a constant *χ* interaction parameter, describing a binodal, spinodal, tie line, and critical point. Then, the morphologies of the blends composed of highly disordered PTB7-Th and crystallizable ITIC were investigated by atomic force microscopy. Subsequently, the surface polarities of the PTB7-Th:ITIC thin films were examined by water contact-angle goniometer, exhibiting a transition at the composition of ~ 60 ± 10 wt.% ITIC. Furthermore, X-ray diffraction indicated the presence of ITIC’s crystallites at ≥ 70 wt.% ITIC. Hence, the PTB7-Th:ITIC system was observed to undergo a phase transition at ~ 60–70 wt.% ITIC.

## Introduction

Conjugated polymers have semiconducting and metallic properties depending on doping concentration, which was first demonstrated using polyacetylene (PA) in 1977^1^. In this PA, carbon atom has one unpaired π-electron based on sp^2^p_z_ hybridization. However, the Peierls instability makes two carbons group together in a one-dimensional chain, resulting in bond alternation and energy gap^2^. Thus, these π-bonded macromolecules such as PA could be used as an active semiconductor for photonic devices such as light-emitting diode, photodetector, and photovoltaic (PV) cell^3^. In addition, just like saturated polymers, they have advantages in both processibility and mechanical properties^4^. However, the low dielectric constants (~ 3–4) of π-bonded organic polymer allow them to have only small-radius Frenkel excitons with diffusion length of ~ 5–47 nm under illumination^5–7^. This characteristic suggests that an appropriate nanoscale phase separation should be essential for excitons to dissociate in a photoactive layer, *i.e*., organic semiconductor mixture^8^.

In 1992, ultrafast photoinduced electron transfer in a conducting polymer-fullerene mixture was discovered^9^. After three years, the bulk-heterojunction (BHJ) concept was introduced for dissociating tightly-bound excitons at the donor:accepter (D:A) interfaces in three-dimensional active layer^10,11^. Then, the blends of poly(3-hexylthiophene) (P3HT) and 1-(3-methoxycarbonyl)propyl-1-phenyl[6,6]C_61_ (PC_61_BM) have been extensively studied^12,13^. However, the bandgap of P3HT is relatively wide (~ 1.9 eV), motivating a brand new design of versatile low bandgap polymers for panchromatic absorption^14–19^. This trend has been also applied to the electron acceptors such as n-type polymers and small molecular non-fullerene acceptors (NFAs)^20,21^. Currently, the power conversion efficiency (PCE) of the best organic PV cells is ~ 19.3% in a single-junction configuration, and the corresponding record of all-polymer solar cells is ~ 17.4%, respectively^22–27^.

For understanding the formation process of a phase-separated BHJ architecture, it is essential to study the phase behavior of D:A polymer-NFA or polymer–polymer blends. However, the fact that π-bonded organic semiconductors can absorb light makes it very restricted to study the phase-separation point of the conjugated polymer solutions with typical experimental methods such as cloud-point measurement^28^ and light scattering^29^. Hence, a theoretical approach could be a simple alternative to capturing the phase behavior of light-absorbing polymer solutions^13,14,30–32^. Accordingly, in this study, we constructed theoretically the phase diagrams of ternary solvent/polymer/NFA (or fullerene derivative) systems as a function of molecular weight, temperature, and electron-acceptor species^33–35^. For this purpose, we employed the Flory–Huggins lattice model^36–38^ for the blend system composed of poly[4,8-bis(5-(2-ethylhexyl)-thiophen-2-yl)benzo[1,2-b;4,5-b0]-dithiophene-2,6-diyl-alt-(4-(2-ethylhexyl)-3-fluorothieno[3,4-b]-thiophene-)-2-carboxylate-2-6-diyl] (PTB7-Th) and 3,9-bis(2-methylene-(3-(1,1-dicyanomethylene)-indanone))-5,5,11,11-tetrakis(4-hexylphenyl)-dithieno[2,3-d:2’,3’-d’]-s-indaceno[1,2-b:5,6-b’]dithiophene (ITIC)^19,39–42^. Here, PTB7-Th is a highly disordered polymer whereas ITIC is an easily crystallizable NFA. Firstly, the morphologies of the PTB7-Th:ITIC films were investigated as a function of composition using atomic force microscopy (AFM). Secondly, through the water contact-angle measurement, the phase behavior of the binary films was examined with assumption that a phase-separated sample might display a different surface energy compared to a homogenous one as long as the two components of blends have a different solubility parameter. Finally, through the x-ray diffraction (XRD) patterns, we tried to find out the phase-separation point, e.g., the crystallization of ITIC molecules, of the binary PTB7-Th:ITIC system.

## Materials and methods

### Materials

The π-bonded PTB7-Th (C_49_H_57_FO_2_S_6_)_n_ and ITIC (C_94_H_82_N_4_O_2_S_4_) were purchased from 1-Material, Inc. (Quebec, Canada). Here, PTB7-Th has the number average molecular weight, $M_{n} \approx$ 40 kg/mol, the weight average molecular weight,$M_{w} \approx$ 100 kg/mol, and polydispersity index, ${\text{PDI}} \approx$ 2.5 based on the polystyrene standard. Chlorobenzene (CB) was provided from Sigma-Aldrich, Inc. (Taufkirchen, Germany). All these materials were used as received without further purification.

### Methods

The PTB7-Th:ITIC solutions with concentration of 15 mg/mL in CB were prepared as a function of composition and spincoated at 1500 rpm on a glass slide for studying the morphologies, water-contact angle, and XRD patterns. The morphologies of the PTB7-Th:ITIC films were characterized by the tapping-mode AFM (XE-100 Park Systems, Mannheim, Germany). Here, the AFM data were analyzed using the Park Systems XEI software. The water contact angles ($\theta$) of surfaces were measured using a CAM101 goniometer. The measurements were performed following the sessile drop method. A series of images for the water drop (∼5–8 µL) was acquired over 15 s, during which the 8 measurements were taken for different parts of each sample. An average contact angle for sample was then calculated. XRD was performed using the D8 Advance diffractometer (Bruker, Karlsruhe, Germany) with Cu-Kα cathode (*λ* = 1.54 Å). The Bragg–Brentano geometry measurement was applied in coupled ${{2\theta } \mathord{\left/ {\vphantom {{2\theta } \theta }} \right. \kern-\nulldelimiterspace} \theta }$ mode. The scan rate was 1.2°/min with scanning step 0.02° in range of $2\theta$ = 2° to 60°. Background subtraction, occurring from air scattering and glass substrates, was performed using DIFFRAC.EVA program.

## Results and discussion

**Figure 1:**
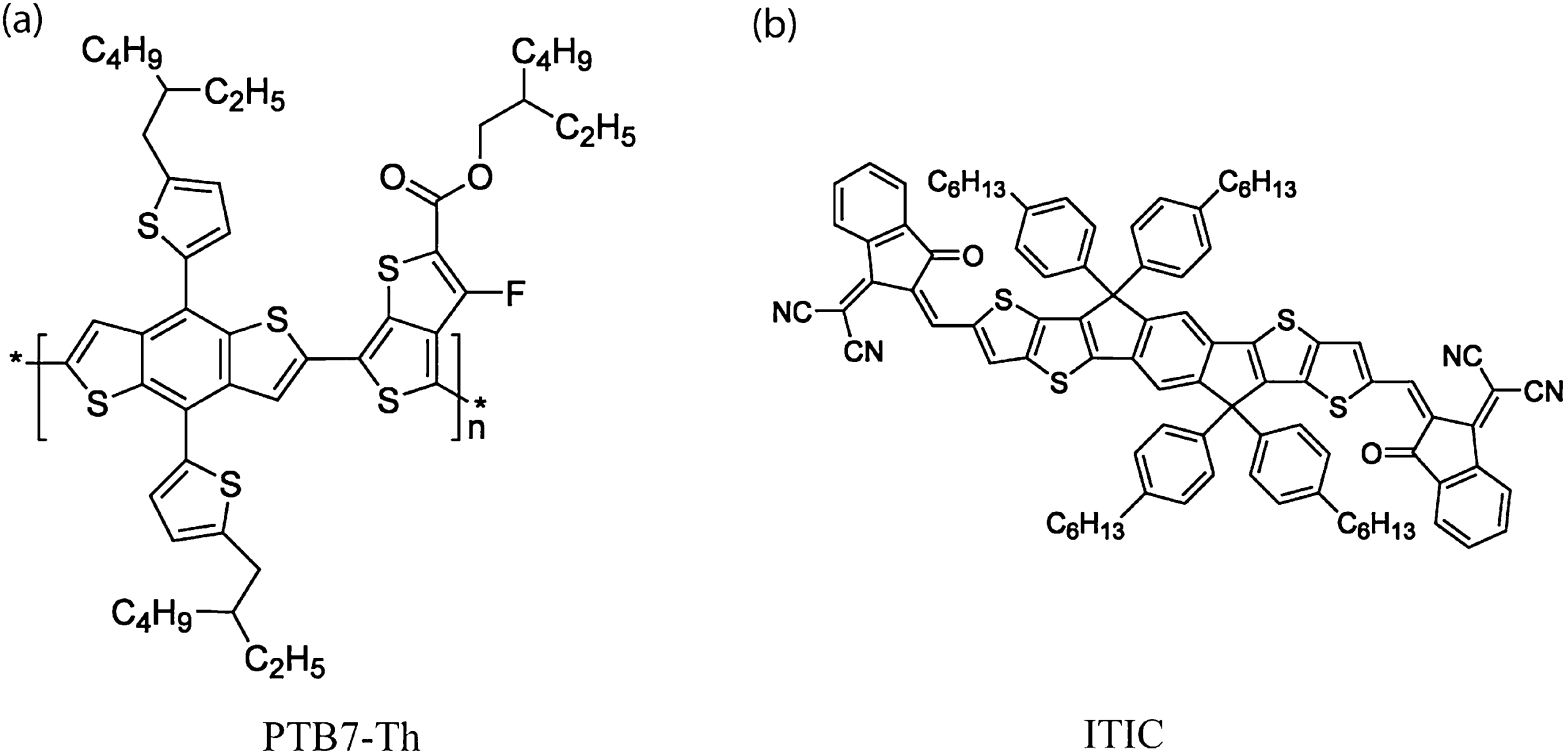
Chemical structures of (**a**) electron-donating polymer (PTB7-Th), and (**b**) electron-accepting small molecule (ITIC).

Figure 1 shows the chemical structures of the model systems, PTB7-Th and ITIC. Specifically, PTB7-Th (also known as PCE10 or PBDTTT-EFT) is a high performance polymer based on two-dimensional (2D) benzodithiophene (BDT) and thieno[3,4-b]thiophene (TT) units^19^. In this study, we investigated the phase behavior of the ternary CB/PTB7-Th/ITIC solution based on the Flory–Huggins lattice model. According to Yilmaz et al.^43^, the Flory–Huggins model for a ternary polymer solution could be expressed as follows^44,45^,

1 $$\frac{{\Delta G_{mix} }}{RT} = n_{1} \ln \phi_{1} + n_{2} \ln \phi_{2} + n_{3} \ln \phi_{3} + \chi_{12} n_{1} \phi_{2} + \chi_{13} n_{1} \phi_{3} + \chi_{23} n_{2} \phi_{3}$$

where $\Delta G_{mix}$ is the molar Gibbs energy of mixing,* R* is the Gas constant, *T* is temperature, $\phi_{i}$ is volume fraction of component *i*, and $n_{i}$ is the number of moles of component $i$. Furthermore,$\chi_{ij} {{ = \hat{V}_{1} } \mathord{\left/ {\vphantom {{ = \hat{V}_{1} } {RT}}} \right. \kern-\nulldelimiterspace} {RT}}\left( {\delta_{i} - \delta_{j} } \right)^{2} + 0.34$ is the intermolecular interaction parameter^46^, in which $\hat{V}_{1}$ is a molar volume of solvent and $\delta_{i}$ or $\delta_{j}$ is a solubility parameter of component $i$ or $j$ (= 1, 2, and 3). Note that in this work, the components 1, 2, and 3 correspond to solvent, polymer, and nonsolvent, respectively. Then, through the equilibrium of the chemical potential, $\Delta \mu_{i}^{\alpha } = \Delta \mu_{i}^{\beta } \quad \left( {i = 1,\;2,\;3} \right)$, the binodal curve could be calculated for ternary systems^30^. Here, $\Delta \mu_{i}$ is defined as ${{\partial \Delta G_{mix} } \mathord{\left/ {\vphantom {{\partial \Delta G_{mix} } {\partial n_{i} }}} \right. \kern-\nulldelimiterspace} {\partial n_{i} }}$, and the spinodal curve and critical point could be obtained from the second and third derivatives of $\Delta G_{mix}$, respectively, according to our previous works^30–32^ (see supplementary information for details). Here, the parameters used for this theoretical work were summarized in Table 1. Note that the molar volume ($v_{i} = {{MW_{i} } \mathord{\left/ {\vphantom {{MW_{i} } {\rho_{i} }}} \right. \kern-\nulldelimiterspace} {\rho_{i} }}$) could be calculated from the ratio of molecular weight and density. For example, PTB7-Th has $v_{2}$ = {(300,000 g/mol)/(1.15 g/cm^3^)} = 260,870 cm^3^/mol and ITIC has $v_{3}$ = {(1428 g/mol) /(1.24 g/cm^3^)} = 1152 cm^3^/mol, respectively.

**Table 1:** Solubility parameter ($\delta_{i}$), molecular weight (MW), molar volume ($v_{i}$), density ($\rho$), chemical structure and reference for materials. Here, MW is *M*_*n*_ in the case of PTB7-Th.

Materials	$\delta_{i}$^a^ (cal/cm^3^)^1/2^	$\delta_{i}$ (MPa^1/2^)	MW (g/mol)	$v_{i}$(cm^3^/mol)	$\rho$ (g/cm^3^)	Chemical structure	Refs.
PTB7-Th	9.3	18.98	300,000	260,870	1.15	(C_49_H_57_FO_2_S_6_)_n_	^47,48^
ITIC	11.8	24.18	1428	1152	1.24	C_94_H_82_N_4_O_2_S_4_	^48,49^
PC_61_BM	11.3	23.15	910	607	1.5	C_72_H_14_O_2_	^13,14,30^
PC_71_BM	11.2	22.95	1031	687	1.5	C_82_H_14_O_2_	^13,14,30^
CB	9.5	19.47	112.56	101.41	1.11	C_6_H_5_Cl	^13,14,30^

^a^Note that this solubility parameter with dimension of (cal/cm^3^)^1/2^ is used for Eq. (2).

**Figure 2:**
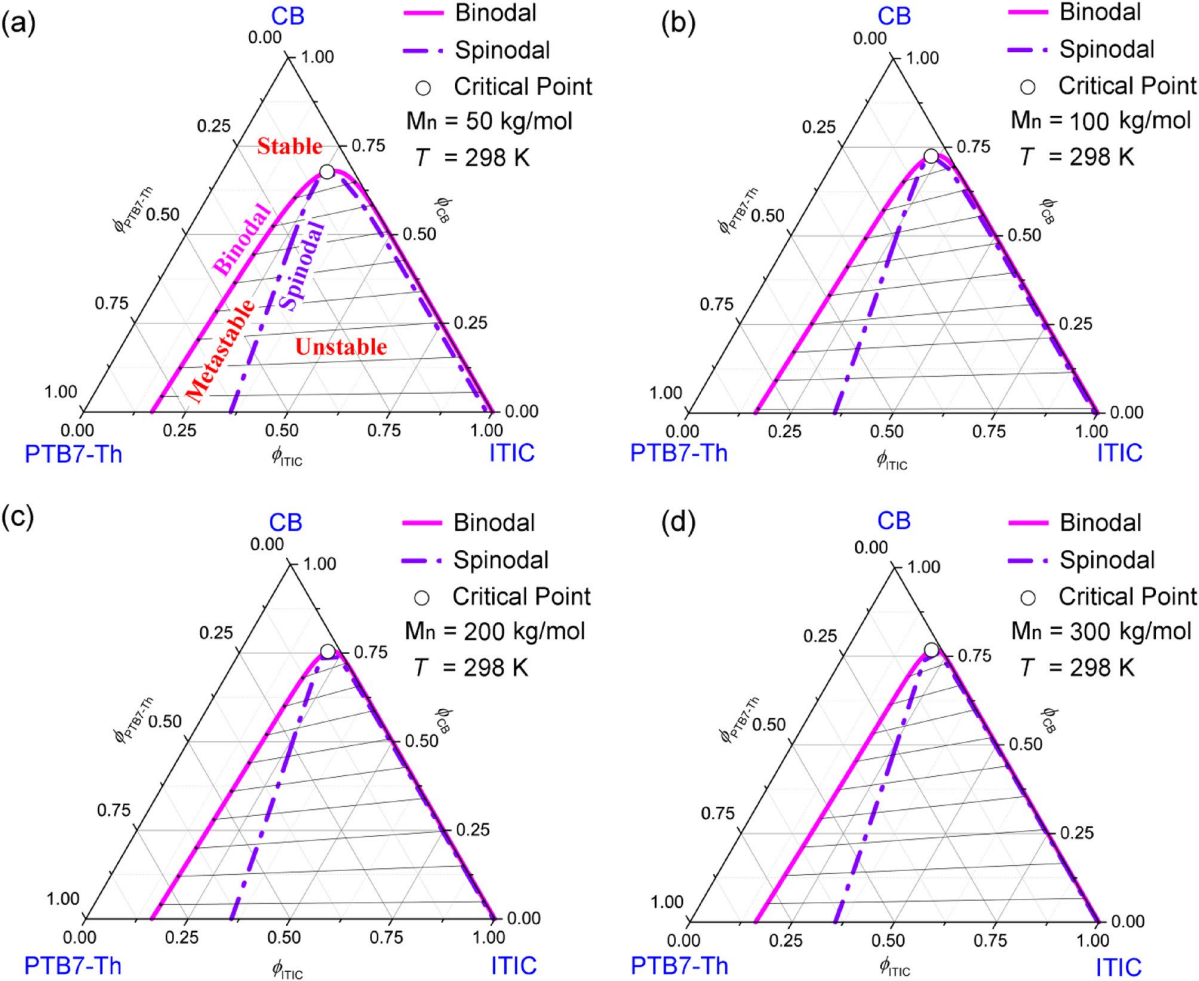
Phase diagrams for the ternary CB/PTB7-Th/ITIC system as a function of molecular weight at *T* = 298 K. $\chi_{12} = 0.35$, $\chi_{13} = 1.25$, and $\chi_{23} = 1.41$. (**a**) *M*_*n*_ = 50 kg/mol: *s* = 0.002332 and *r* = 0.087923. (**b**) *M*_*n*_ = 100 kg/mol: *s* = 0.001127 and *r* = 0.087923. (**c**) *M*_*n*_ = 200 kg/mol: *s* = 0.000583 and *r* = 0.087923. (**d**) *M*_*n*_ = 300 kg/mol: *s* = 0.000389 and *r* = 0.087923.

Figure 2 shows the ternary isothermal phase diagrams of the CB/PTB7-Th/ITIC system as a function of the number average molecular weight $M_{n}$ (physically, equivalent to chain length) at *T* = 298 K. Table 2 exhibits the parameters used for calculating these phase diagrams. As shown in Fig. 2, the critical point may shift up from the axis (PTB7-Th and ITIC) with increasing $M_{n}$, indicating the liquid–liquid (L–L) demixing is more favorable in high $M_{n}$ polymer. Notably, the critical points $\left( {\phi_{1c} ,\;\phi_{2c} ,\;\phi_{3c} } \right)$ are (0.6774, 0.0659, 0.2566) at 50 kg/mol, (0.7249, 0.0443, 0.2307) at 100 kg/mol, (0.7543, 0.0311, 0.2145) at 200 kg/mol, and (0.7676, 0.0251, 0.2071) at 300 kg/mol, respectively. Hence, as increasing $M_{n}$, one phase solution can be easily phase-separated at diluted concentration. Here, the demixing gap is defined by binodal curve, indicating L-L phase transition. Furthermore, the gap area between binodal and spinodal curve is a metastable region whereas the area under the spinodal is an unstable region. When a solution passes through a spinodal curve into an unstable region, we call it spinodal decomposition^50,51^. Kahn and Hilliard suggested a kinetic model for explaining the spinodal decomposition process as follows^50,51^,

2 $$\frac{\partial c}{{\partial t}} = M\left( {\frac{{\partial^{2} f}}{{\partial c^{2} }}} \right)\nabla^{2} c - 2M\kappa \nabla^{4} c$$

where $c$ is concentration, *t* is time, *M* is a mobility, *f* is a free energy density of homogeneous material of composition *c* and $\kappa$ is a positive parameter, respectively. In polymer solutions, *f* corresponds to $\Delta G_{mix}$^52–54^. Basically, it is Fick’s law of diffusion with diffusivity $D = M\left( {{{\partial^{2} f} \mathord{\left/ {\vphantom {{\partial^{2} f} {\partial c^{2} }}} \right. \kern-\nulldelimiterspace} {\partial c^{2} }}} \right)$. In unstable region, ‘${{\partial^{2} f} \mathord{\left/ {\vphantom {{\partial^{2} f} {\partial c^{2} }}} \right. \kern-\nulldelimiterspace} {\partial c^{2} }} < 0$ → $\;\;D < 0$’ indicates an uphill diffusion, resulting in (1) a spontaneous phase separation without nucleation, and (2) connectivity of the D:A phases fitting for interpenetrating BHJ structure for polymer PV devices. On the other hand, in a metastable region, ‘ ${{\partial^{2} f} \mathord{\left/ {\vphantom {{\partial^{2} f} {\partial c^{2} }}} \right. \kern-\nulldelimiterspace} {\partial c^{2} }} > 0$ → $\;\;D > 0$’ denotes a down-hill diffusion, where nucleation process needs a work (*W*) as follows^55^,

3 $$W = 4\pi r^{2} \gamma - \frac{4}{3}\pi r^{3} \Delta P$$

where $\gamma$ is the surface tension at the interface, *r* is the radius of nucleus, and $\Delta P$ is the hydrostatic pressure requiring to maintain nucleus in equilibrium with the exterior phase. Through the first derivative of Eq. (3), we may obtain a critical radius, $r_{crit} = {{2\gamma } \mathord{\left/ {\vphantom {{2\gamma } {\Delta P}}} \right. \kern-\nulldelimiterspace} {\Delta P}}$. Hence, the minimum work ($W_{\min }$) is ${4 \mathord{\left/ {\vphantom {4 3}} \right. \kern-\nulldelimiterspace} 3} \cdot \pi r_{crit}^{2} \gamma$, indicating that a nucleus can grow when $r > r_{crit}$ otherwise it collapses. At this moment, it is noteworthy that above two phase separation mechanism (i.e., spinodal decomposition and nucleation-and-growth) are amorphous-amorphous (or L-L) phase separation. However, as shared with insulating polymers (*i.e*., saturated hydrocarbons with sp^3^ hybridization), not only amorphous phase separation, but also crystallization could proceed simultaneously in polymer solutions. Specifically, in the PTB7-Th:ITIC system, ITIC is highly crystallizable, indicating that the crystallization of ITIC could be another route for phase separation.

**Table 2:** Flory–Huggins interaction parameters and molar volume ratios for the ternary systems: PTB7-Th’s *M*_*n*_ = 300 kg/mol.

Ternary system	Flory–Huggins interaction parameter			Molar volume ratio	
	$\chi_{12}$	$\chi_{13}$	$\chi_{23}$	$s = {{v_{1} } \mathord{\left/ {\vphantom {{v_{1} } {v_{2} }}} \right. \kern-\nulldelimiterspace} {v_{2} }}$	$r = {{v_{1} } \mathord{\left/ {\vphantom {{v_{1} } {v_{3} }}} \right. \kern-\nulldelimiterspace} {v_{3} }}$
CB/PTB7-Th/ITIC	2.0 K/*T* + 0.34	270.0 K/*T* + 0.34	319.0 K/*T* + 0.34	0.000389	0.088030
CB/PTB7-Th/PC_61_BM	2.0 K/*T* + 0.34	165.4 K/*T* + 0.34	204.2 K/*T* + 0.34	0.000389	0.167068
CB/PTB7-Th/PC_71_BM	2.0 K/*T* + 0.34	147.5 K/*T* + 0.34	184.2 K/*T* + 0.34	0.000389	0.147613

**Figure 3:**
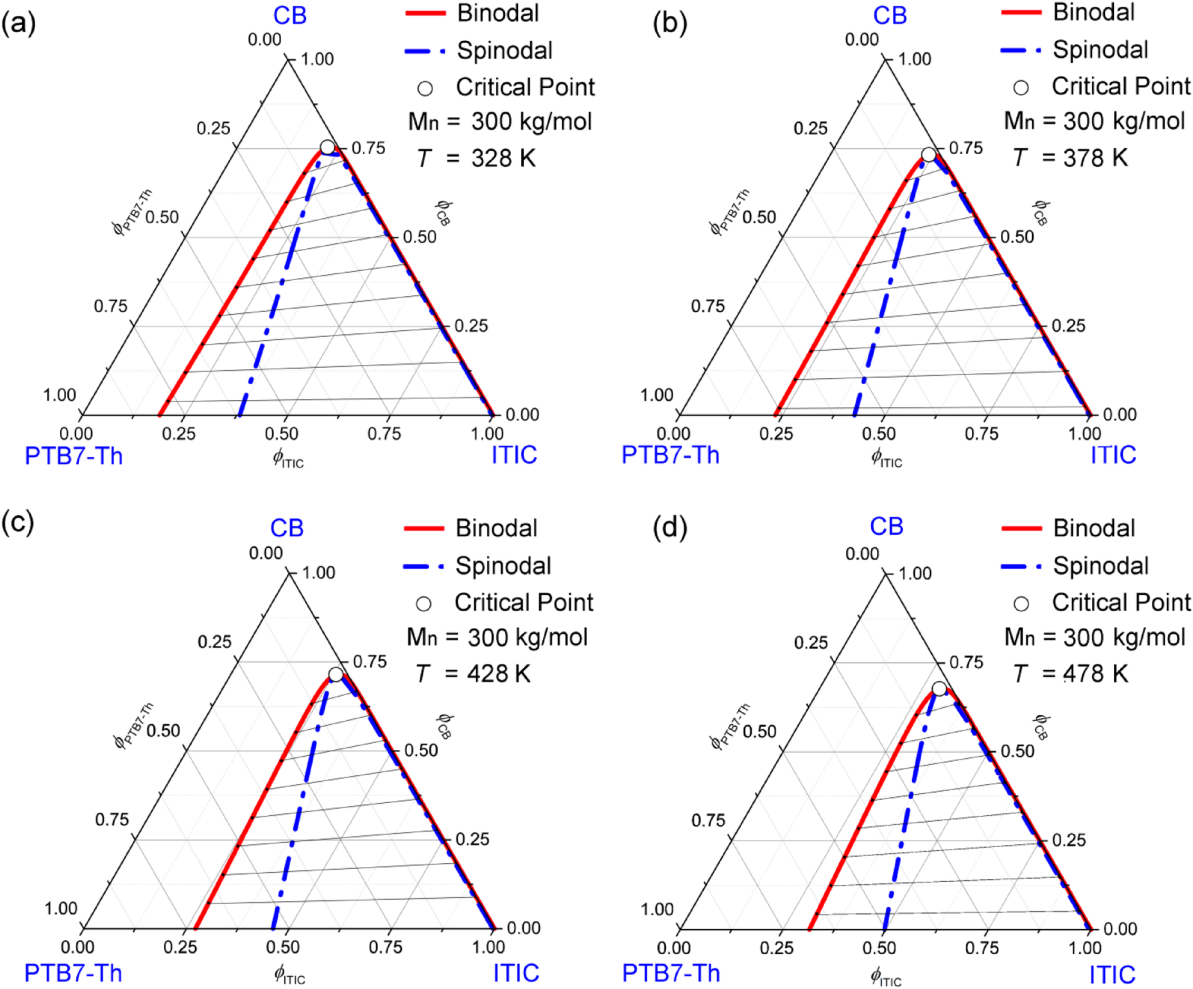
Phase diagrams for the ternary CB/PTB7-Th/ITIC system as a function of temperature: PTB7-Th’s *M*_*n*_ = 300 kg/mol. (**a**) *T* = 328 K: $\chi_{12} = 0.35$, $\chi_{13} = 1.16$, and $\chi_{23} = 1.31$. (**b**) *T* = 378 K: $\chi_{12} = 0.35$, $\chi_{13} = 1.05$, and $\chi_{23} = 1.18$. (**c**) *T* = 428 K: $\chi_{12} = 0.35$, $\chi_{13} = 0.97$, and $\chi_{23} = 1.09$. (**d**) *T* = 478 K: $\chi_{12} = 0.34$, $\chi_{13} = 0.91$, and $\chi_{23} = 1.01$. Here, PTB7-Th’s *M*_*n*_ = 300 kg/mol, *s* = 0.000389, and *r* = 0.087923.

Figure 3 shows the ternary phase diagrams of the CB/PTB7-Th/ITIC system as a function of temperature. Here, by increasing temperature, the critical points were shifted down toward the PTB7-Th‒ITIC axis, indicating the upper critical solution temperature (UCST) phase behavior. Importantly, the temperature-induced phase separation (TIPS) is a typical process for a membrane formation based on the UCST phase behavior. At this moment, it is noteworthy that although π-bonded semiconducting polymer and saturated insulating polymer have different hybridization such as sp^2^p_z_ vs. sp^3^, the origin of phase separation such as TIPS, immersion precipitation, and others could be shared each other, indicating that the rich knowledge accumulated in the field of insulating polymers^56,57^ could be utilized for π-bonded semiconducting and metallic polymers.

**Figure 4:**
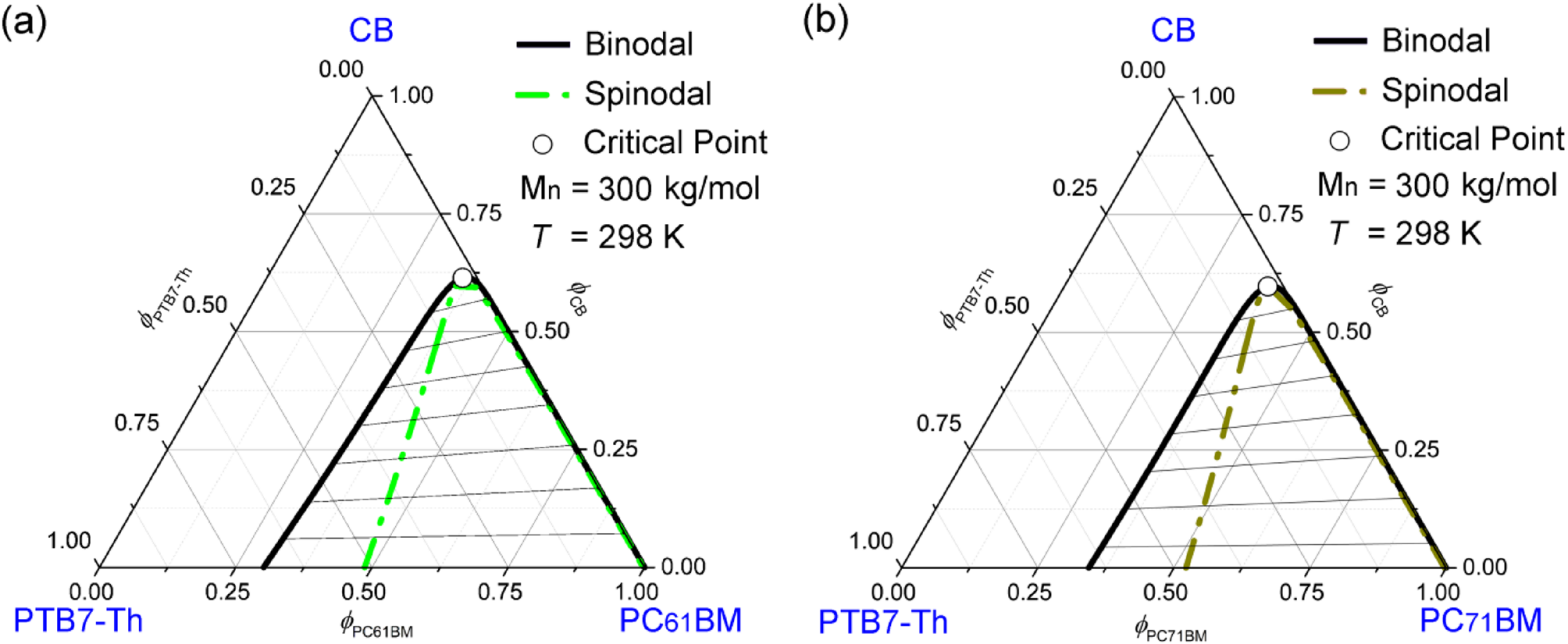
Phase diagrams for the ternary CB/PTB7-Th/Fullerene system as a function of electron acceptors: PTB7-Th’s *M*_*n*_ = 300 kg/mol and *T* = 298 K. (**a**) CB/PTB7-Th/PC_61_BM:$\chi_{12} = 0.35$, $\chi_{13} = 0.90$, $\chi_{23} = 1.03$, *s* = 0.000389, and *r* = 0.167068. (**b**) CB/PTB7-Th/PC_71_BM:$\chi_{12} = 0.35$, $\chi_{13} = 0.84$, $\chi_{23} = 0.96$, *s* = 0.000389, and *r* = 0.147613.

Figure 4 shows the ternary phase diagrams of (a) CB/PTB7-Th/PC_61_BM and (b) CB/PTB7-Th/PC_71_BM systems for examining the effect of electron acceptors on the phase behavior of polymer solutions. These phase diagrams in Fig. 4 could be compared with that of CB/PTB7-Th/ITIC (at *T* = 298 K and *M*_*n*_ = 300 kg/mol) in Fig. 2d. As shown in Fig. 4, the fullerene derivatives (PC_61_BM and PC_71_BM) have a better miscibility with PTB7-Th than the non-fullerene ITIC by showing a smaller demixing gap under each spinodal curve. Furthermore, PC_71_BM is more miscible with PTB7-Th than PC_61_BM in the PTB7-Th/CB solutions. Table 2 summarizes the parameters used for this comparison.

**Figure 5:**
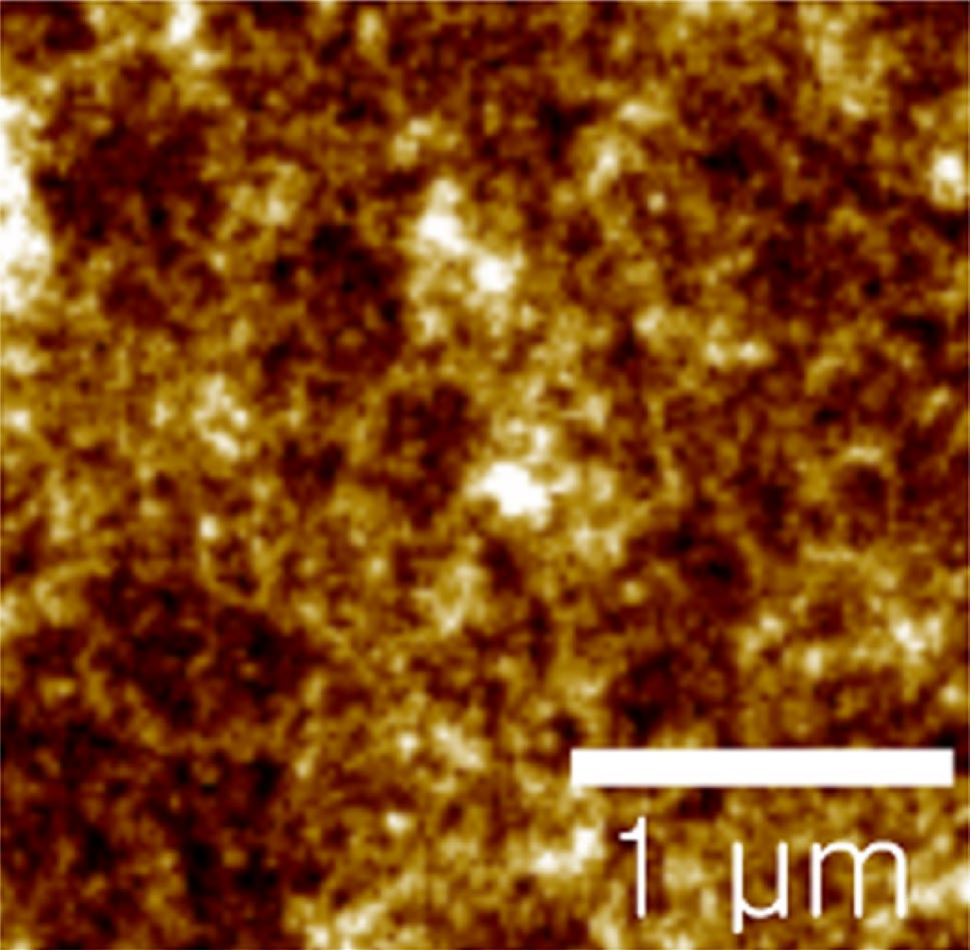
Tapping mode AFM height images of a pure PTB7-Th film.

Until now, we investigated phase behavior of ternary polymer solutions based on Flory–Huggins theory. Now, let us examine it using experimental tools such as AFM, water contact-angle and XRD. Figure 5 shows the tapping mode AFM height image of a pure PTB7-Th film, exhibiting a highly disordered morphology with average surface roughness (~ 0.65 nm) and root-mean-square roughness (~ 0.914 nm). On the other hand, its corresponding phase image shows uniformity within the instrumental resolution of this AFM (see Figure S1 in Supplementary Information). In addition, we investigated the topographies of each blend film as a function of composition (Figure S2). However, in our study, by the AFM image alone, it was hard to interpret the phase behavior of PTB7-Th:ITIC film samples. Hence, we rely on other experimental methodologies such as XRD and water contact-angle measurement.

**Figure 6:**
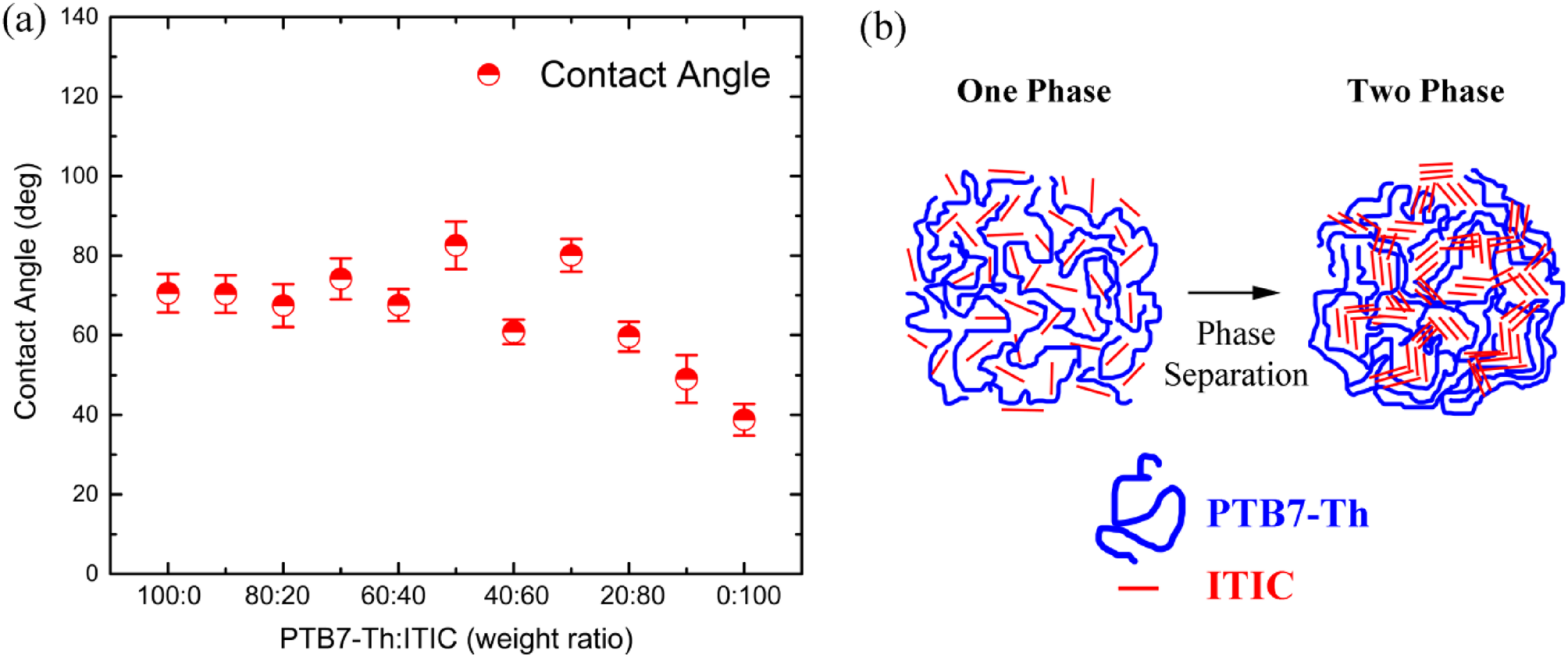
(**a**) Water contact angle for the PTB7-Th:ITIC films as a function of weight ratio of components. (**b**) The phase-separation process from one phase to two phase in the PTB7-Th:ITIC films by increasing weight fraction of ITIC.

Figure 6a shows the water contact-angle data as a function of composition, which was carried out to examine the surface energy depending on the microstructures of a film as our previous studies^30,32^. Note that the data is an average value by measuring eight different spots of a film. As shown in Fig. 6a, the overall observation is that the contact angle change at ~ 60 ± 10 wt.% ITIC, in which a little fluctuation in data may contain an experimental uncertainty in this water contact-angle experiment. Second, when the composition is greater than or equal to ~ 70 wt.% ITIC, the water contact angle decreases linear, indicating the enhancement of hydrophilicity of thin films. This is because ITIC with *δ* = 11.8 (cal/cm^3^)^1/2^ is more polar than PTB7-Th with *δ* = 9.3 (cal/cm^3^)^1/2^ (see Table 1). Importantly, to the best of authors’ knowledge, this is the first detailed report demonstrating that the contact-angle measurement could be a useful tool for identifying the phase separation of π-bonded polymer blends. Figure 6b shows a schematic expression regarding a phase separation process in the PTB7-Th:ITIC films. When there is small amount of ITIC in the binary blend film, ITIC may dissolve into the free volume of PTB7-Th, forming a solid solution. However, when the composition of PTB7-Th:ITIC is ~ 60 ± 10 wt. % ITIC, the ITIC molecules may be phase-separated out. Note that if the water contact-angle data were not related with the phase-separation morphology but with a simple composition only (PTB7-Th:ITIC wt. ratio), it would decrease linearly from 70.51 ± 4.84° (PTB7-Th) to 38.80 ± 3.99° (ITIC). However, as shown in Fig. 6a, the data trend is not linear, but displays a drastic change at ~ 60 ± 10 wt. % ITIC, suggesting a phase separation as schematically explained in Fig. 6b.

**Figure 7:**
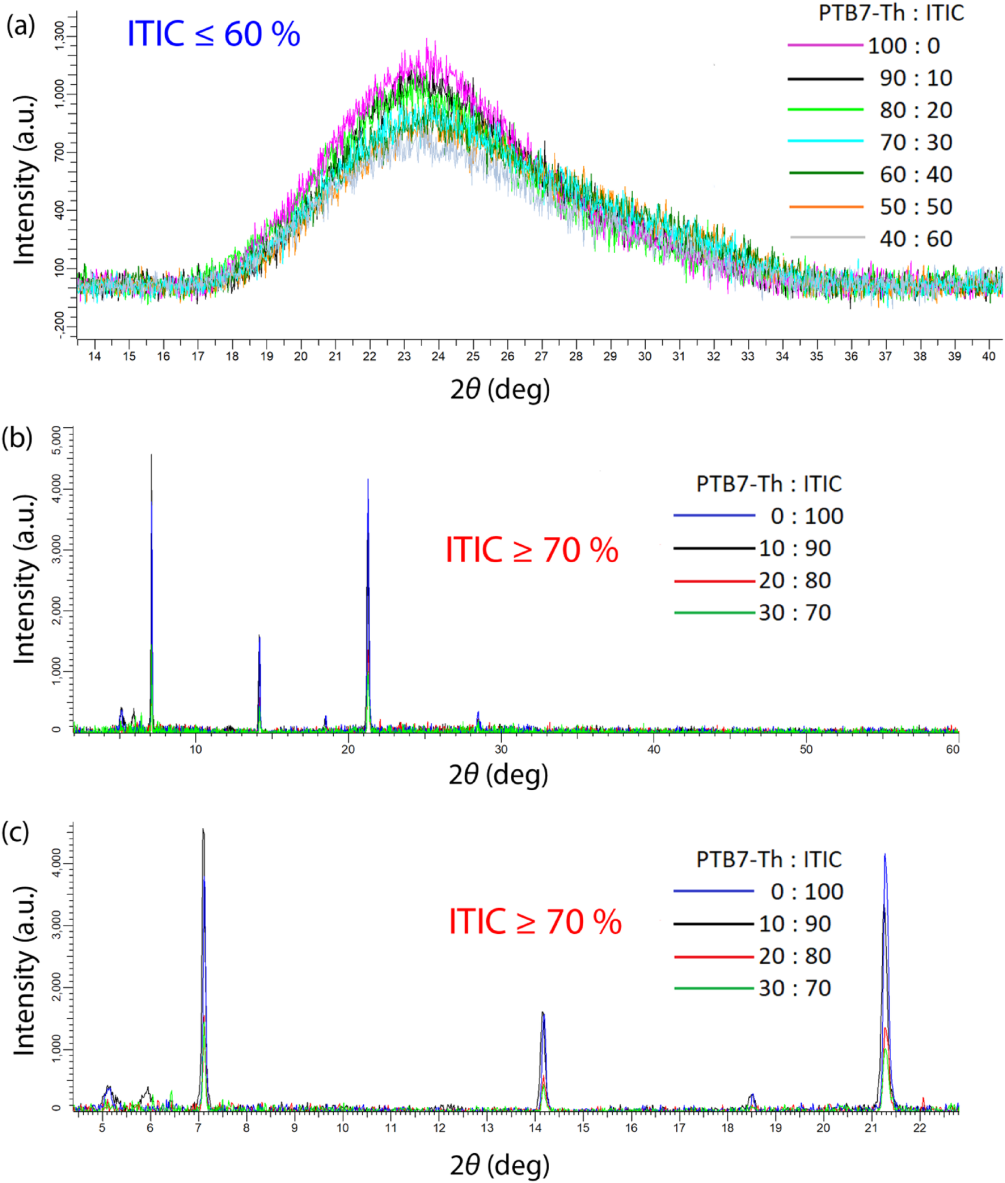
XRD patterns of PTB7-Th:ITIC films as a function of weight ratio. (**a**) PTB7-Th:ITIC = 100:0, 90:10, 80:20, 70:30, 60:40, 50:50 and 40:60. (**b**) PTB7-Th:ITIC = 30:70, 20:80, 10:90, and 0:100. (**c**) Magnified XRD pattern when PTB7-Th:ITIC = 30:70, 20:80, 10:90, and 0:100.

Figure 7 shows the XRD pattern as a function of composition. When the composition of the PTB7-Th:ITIC blends is in the range of 10 to 60 wt.% ITIC, the XRD patterns are overlapped with a pure PTB7-Th polymer (i.e., 0 wt.% ITIC) as shown in Fig. 7a. Here, it is notable that the highly disordered PTB7-Th is known to be at the boundary of amorphous and semicrystalline^39^. However, in this study, according to the XRD data in Fig. 7a, PTB7-Th is simply amorphous, displaying a typical amorphous halo. In general, amorphous polymers have no long range order except for a short one although there were some reports claiming locally ordered regions in an amorphous polymer^58,59^. Interestingly, when the composition is in the range of 70 to 100 wt.% ITIC (Fig. 7b and c), the sharp crystallite peaks were observed, indicating that ITIC was phase-separated out through crystallization. Notably, the crystallite size (*t*) was estimated through the Scherrer equation $t = {{K \cdot \lambda } \mathord{\left/ {\vphantom {{K \cdot \lambda } {\left( {\beta \cdot \cos \theta } \right)}}} \right. \kern-\nulldelimiterspace} {\left( {\beta \cdot \cos \theta } \right)}}$, in which *K* is 0.98, $\lambda$ is 0.154 nm, and $\beta$ is a full width at half maximum (FWHM) at angle, $2\theta \approx 21.3^\circ$. Resultantly, when the compositions of PTB7-Th:ITIC blends were 30:70, 20:80, 10:90, and 0:100 (weight ratio), the estimated *t* was 65.5 nm, 68.8 nm, 77.2 nm, and 68.7 nm, respectively (see Table 3). Hence, the average crystallite size is ~ 70.05 ± 5.01 nm. However, if we calculate the amorphous halo based on the same Scherrer equation, the *t* value would be 1.3 nm when $\beta$ = 0.118682 and $2\theta \approx 23.5^\circ$, indicating the peak is an amorphous halo as expected. Note that ITIC single crystal was reported to have lattice parameters of **a** = 14.88 Å, **b** = 15.47 Å, **c** = 18.08 Å, *α* = 99.27°, *β* = 101.50°, and *γ* = 108.37°^60^. Thus the estimated *t* value is less than any lattice parameter of ITIC, suggesting that the PTB7-Th:ITIC blends (ITIC $\le$ 60 wt.%) are in amorphous state as shown in Fig. 7a. Finally, considering that, through the XRD patterns, we can examine only crystallization as an evidence of phase separation (i.e., liquid–solid phase transition), the ternary phase diagrams in Figs. 2, 3, and 4 displaying liquid–liquid phase transition (such as spinodal decomposition in an unstable region and nucleation-and-growth in a metastable region) should be important for understanding the phase behavior of PTB7-Th based blends and solutions, at least qualitatively. Remind that two different phase separation (i.e., amorphous L-L and crystallization) may proceed simultaneously^56,57^ in polymer solutions with a crystallizable component, suggesting that it is important to understand ternary phase diagrams in Figs. 2, 3, and 4 based on Flory–Huggins theory.

**Table 3:** Crystallite size ($t$) of PTB7-Th: ITIC blends at $2\theta \approx 21.3^\circ$.

	PTB7-Th:ITIC (wt. ratio)			
	30:70	20:80	10:90	0:100
$\beta$(radian)	0.00234	0.00223	0.00199	0.00224
$t$(nm)	65.5	68.7	77.2	68.7

## Conclusion

In summary, the phase behavior of the amorphous/crystalline PTB7-Th:ITIC blends was studied. First, the phase diagrams of ternary PTB7-Th:ITIC solutions were constructed based on the classical Flory–Huggins theory, capturing an essential L-L phase transition. In this work, it was found that the fullerene derivatives (PC_61_BM and PC_71_BM) were more miscible with PTB7-Th than non-fullerene acceptor (ITIC) by showing a diminished demixing gap. Interestingly, the water contact-angle data showed a surface-polarity transition at ~ 60 ± 10 wt.% ITIC whereas the XRD patterns displayed a clear evidence of ITIC crystallization at ≥  ~ 70 wt.% ITIC. Hence, based on the contact angle and XRD data, it was found that the binary PTB7-Th:ITIC system should undergo a phase transition at ~ 60–70 wt.% ITIC. As a future work, the phase-separation dynamics should be a topic of interests for understanding the correlation between π-bonded polymer processing and morphologies, affecting material properties and optoelectronic device performances. Here it is noteworthy that a thin-film process (e.g., spin-coating) in organic PVs is a non-equilibrium one, indicating a polymer:NFA system may undergo a further phase transformation (separation) to reach equilibrium for reducing Gibbs free energy although its kinetics is unknown, which is related with a stability of organic solar cells.

## Supplementary Information

Supplementary Information.

## Data Availability

The datasets used and/or analyzed during the current study available from the corresponding author on reasonable request.
