# Supplementary material for: Phase behavior of π-conjugated polymer and non-fullerene acceptor (PTB7-Th:ITIC) solutions and blends
Source: Sci Rep. 2022 Dec 2;12:20849. doi: 10.1038/s41598-022-25476-9 (PMC9718827; doi:10.1038/s41598-022-25476-9)
Supplement: Supplementary file 1 — Supplementary Information. [file 41598_2022_25476_MOESM1_ESM.docx]

Supplementary Information

Phase Behavior of π-Conjugated Polymer and Non-Fullerene Acceptor (PTB7-Th/ITIC) Solutions and Blends

Jung Yong Kim^1,2^,*, Pawel Jarka^3^, Barbara Hajduk^4^, Henryk Bednarski^4^, Urszula Szeluga^4^ & Tomasz Tański^3^

^1^ Department of Materials Science and Engineering, Adama Science and Technology University, P.O. Box 1888, Adama, Ethiopia

^2^ Center of Advanced Materials Science and Engineering, Adama Science and Technology University, P.O. Box 1888, Adama, Ethiopia

^3^ Institute of Engineering Materials and Biomaterials, Faculty of Mechanical Engineering, Silesian University of Technology, 44-100 Gliwice, Poland

^4^ Centre of Polymer and Carbon Materials, Polish Academy of Sciences, M. Curie-Skłodowska 34 Street, 41-819 Zabrze, Poland

* Corresponding author

E-mail: jungyong.kim@astu.edu.et

ORCID: Jung Yong Kim: 0000-0002-7736-6858

***A. Estimation of solubility parameter:***

The solubility parameter (δ) was estimated from the water contact angle (***θ***) data. According to **Li and Neumann,^S1,S2^ the *θ* value is a function of surface energy,**

$\cos\theta=-1+2\sqrt{\frac{\gamma_{sv}}{\gamma_{lv}}}e^{-\beta\left(\gamma_{lv}-\gamma_{sv}\right)^{2}}$ (1)

where γ_lv_, γ_sv_, and γ_sl_ are surface energies for liquid-vapor, solid-vapor and solid-liquid respectively, and the constant *β* is 0.000115 (m^2^/mJ)^2^. Then, to find a root solution for eq. 1, the **Newton-Rapson method was employed as follows,**

$x_{n+1}=x_{n}-\frac{f\left(x_{n}\right)}{f'\left(x_{n}\right)}\text{ }\left(n=1,2,\cdots\right)$ (2)

**where $f\left(x_{n}\right)=x_{n}-ae^{b\left(1-x_{n}^{2}\right)^{2}};\text{ }a=\left(\cos\theta+1\right)/2,\text{ }b=\beta\gamma_{lv}^{2},\text{ }x^{2}=\gamma_{sv}/\gamma_{lv};\text{ }\gamma_{lv}\text{ = }72.8mJ/m^{2}$for water. Then, by calculating the** solid-vapor **surface energy (*γ*_sv_), the solubility parameter (**$\delta$) could be estimated based on the below relation,

$\delta\left[\left(cal/cm^{3}\right)^{1/2}\right]=1.829058\times\sqrt{\gamma_{sv}}$ (3)

where the conversion factor (1.829058) originated from the literature report.^S3^

***B. Calculation of Ternary Phase Diagrams:***

According to Yilmaz *et al*,^S4^ the chemical potential ($\Delta\mu_{i}=\partial\Delta G_{mix}/\partial n_{i}$) of component *i*, could be expressed as follows,^S5,S6^

(4)

(5)

(6)

where $s=\nu_{1}/\nu_{2}$, $r=\nu_{1}/\nu_{3}$, and $s/r=\nu_{3}/\nu_{2}$. Here, $\nu_{1}$, $\nu_{2}$ and $\nu_{3}$ are molar volume of components 1, 2 and 3, respectively. Then at the equilibrium condition between α and β phases, we may calculate the binodal curve,

$\Delta\mu_{1}^{\alpha}=\Delta\mu_{1}^{\beta}\text{ }\left(i=1, 2, 3\right)$ (7)

where $\alpha$ and $\beta$ indicate a polymer-lean phase and a polymer-rich phase, respectively. Furthermore, the spinodal curve could be obtained if the below criteria were satisfied,

$\left|G\right|=\left|\begin{matrix} G_{22} & G_{23} \\ G_{32} & G_{33} \end{matrix}\right|=0$ (8)

where . Note that $\Delta\bar{G}_{mix}$ is Gibbs free energy of mixing with unit volume basis, $\nu_{ref}$ is molar volume of the reference component ($=v_{1}$), and$G_{23}G_{33}=\left(G_{23}\right)^{2}$. Finally, at the critical point, the below relation should be satisfied,

(9)

where , , and are the volume fractions of component 1, 2, and 3 at critical point, respectively. Then, we are ready to calculate the ternary phase diagrams when the five parameters ($\chi_{12}$, $\chi_{13}$, $\chi_{23}$, $s$, and $r$) were specified according to each ternary system.

***C. Morphologies of PTB7-Th and PTB7-Th:ITIC blend films by Atomic Force Microscopy***


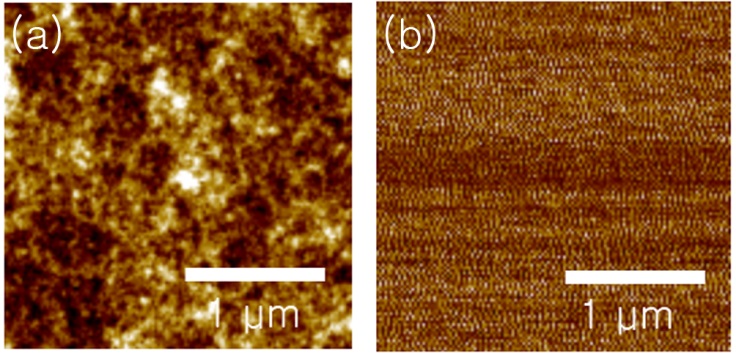


Figure S1. Tapping-mode AFM images of pure PTB7-Th (a) height and (b) phase images.

**
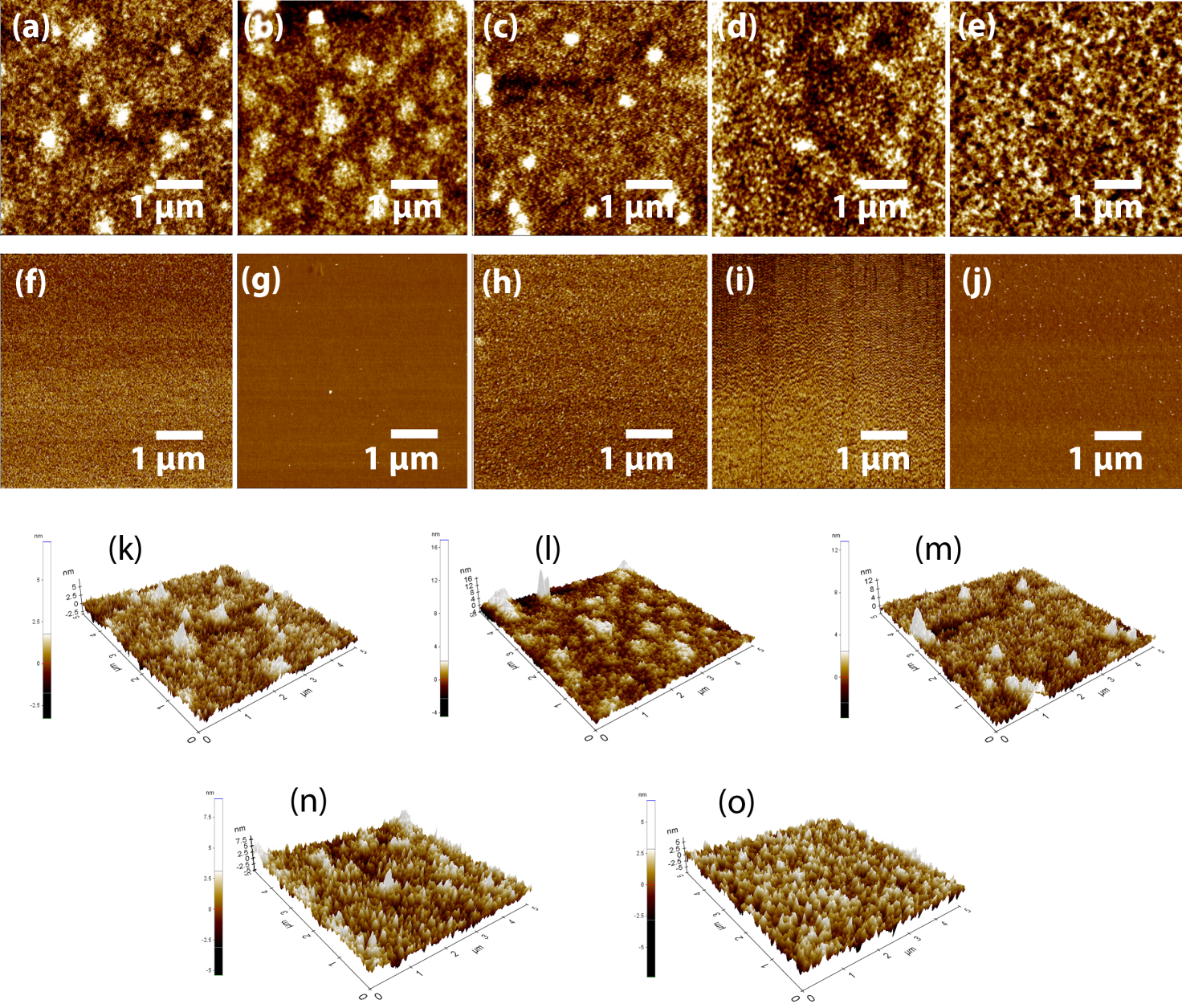
**

Figure S2. Tapping-mode AFM height images: Height images of PTB7-Th:ITIC blend films with ratio of (a) 90:10, (b) 70:30, (c) 50:50, (d) 30:70, and (e) 10:90. Phase images of PTB7-Th:ITIC blend films with ratio of (f) 90:10, (g) 70:30, (h) 50:50, (i) 30:70, and (j) 10:90. Surface 3D images of PTB7-Th:ITIC blend films with ratio of (k) 90:10, (l) 70:30, (m) 50:50, (n) 30:70, and (o) 10:90.

**References**

S1. Li, D. & Neumann, A. W. A Reformulation of the Equation of State for Interfacial Tensions. *J. Colloid Interface Sci*. **137**, 304-307 (1990).

S2. Li, D. & Neumann, A. W. Contact Angles on Hydrophobic Solid Surfaces and Their Interpretation. *J. Colloid Interface Sci*. **148**, 190-200 (1992).

S3. Nilsson, S., Bernasik, A., Budkowski, A. & Moons, E. Morphology and Phase Separation of Spin-Coated Films of Polyfluorene/PCBM Blends. *Macromolecules* **40**, 8291-8301 (2007).

S4. Yilmaz, L. & McHugh, A. J. Analysis of nonsolvent–solvent–polymer phase diagrams and their relevance to membrane formation modeling. *J. Appl. Polym. Sci*. **31**, 997–1018 (1986).

S5. Altena, F.W. & Smolders, C.A. Calculation of liquid-liquid phase separation in a ternary system of a polymer in a mixture of a solvent and a nonsolvent. *Macromolecules* **15**, 1491–1497 (1982).

S6. Hsu, C. C. & Prausnitz, J. M. Thermodynamics of Polymer Compatibility in Ternary Systems. *Macromolecules* **7**, 320–324 (1974).
